# Vibrio Pathogenicity Island-1: The Master Determinant of Cholera Pathogenesis

**DOI:** 10.3389/fcimb.2020.561296

**Published:** 2020-10-06

**Authors:** Ashok Kumar, Bhabatosh Das, Niraj Kumar

**Affiliations:** ^1^Translational Health Science and Technology Institute, Faridabad, India; ^2^Centre for Doctoral Studies, Advanced Research Centre, Manipal Academy of Higher Education, Manipal, India

**Keywords:** cholera pathogenesis, mobile genetic elements (MGEs), VPI-1, quorum sensing, toxin co-regulated pilus

## Abstract

Cholera is an acute secretory diarrhoeal disease caused by the bacterium *Vibrio cholerae*. The key determinants of cholera pathogenicity, cholera toxin (CT), and toxin co-regulated pilus (TCP) are part of the genome of two horizontally acquired Mobile Genetic Elements (MGEs), CTXΦ, and Vibrio pathogenicity island 1 (VPI-1), respectively. Besides, *V. cholerae* genome harbors several others MGEs that provide antimicrobial resistance, metabolic functions, and other fitness traits. VPI-1, one of the most well characterized genomic island (GI), deserved a special attention, because (i) it encodes many of the virulence factors that facilitate development of cholera (ii) it is essential for the acquisition of CTXΦ and production of CT, and (iii) it is crucial for colonization of *V. cholerae* in the host intestine. Nevertheless, VPI-1 is ubiquitously present in all the epidemic *V. cholerae* strains. Therefore, to understand the role of MGEs in the evolution of cholera pathogen from a natural aquatic habitat, it is important to understand the VPI-1 encoded functions, their acquisition and possible mode of dissemination. In this review, we have therefore discussed our present understanding of the different functions of VPI-1 those are associated with virulence, important for toxin production and essential for the disease development.

## Introduction

Cholera is an acute gastrointestinal diarrheal disease that is caused by a bacterium, *Vibrio cholerae* (Kaper et al., 1995). The complete genome sequences of clinical and environmental strains of *V. cholerae* revealed that their genome consists of two circular non-homologous chromosomes that carry nearly 3,900 open reading frames (ORFs). Both the chromosomes of *V. cholerae* consist of core and acquired genomes. The acquired genome of *V. cholerae* harbor several mobile genetic elements (MGEs) and linked with DNA mobility genes and other metabolic functions (Mutreja et al., 2011). Almost all the 7th pandemic *V. cholerae* strains harbor four pathogenicity islands namely (i) Vibrio pathogenicity island-1 (VPI-1), ~41.3 kb in size (ii) Vibrio pathogenicity island-2 (VPI-2), ~57 kb in size, (iii) Vibrio seventh pandemic island-I (VSP-1), ~16 kb in size, and (iv) Vibrio seventh pandemic island-2 (VSP-2), ~26.9 kb in size (Heidelberg et al., 2000). Virulence functions of cholera pathogens are not endogenous, but they are part of the acquired MGEs. *V. cholerae* strains devoid of CTXΦ or VPI-1, two important MGEs ubiquitously present in the toxigenic strains, are non-toxigenic and can't develop cholera in animal model and human volunteer (Pang et al., 2007). Like other bacterial species MGEs present in the genome of *V. cholerae* have several typical characteristics of horizontally acquired elements such as (i) sporadic distribution (ii) encode DNA recombinases (iii) located in *tRNA/ssrA* or *dif* loci (iv) direct repeat sequences at the borders (v) distinct GC content and (vi) unstable.

In this review, we discuss dynamics of VPI-1; different functions encoded by the VPI-1 linked with its mobility and modulate the virulence cascades. Special focus is given to understand how VPI-1 contributed in the emergence of toxigenic pandemic strains.

## The Pathogen (*V. cholerae*) and Integrative Mobile Genetic Elements (MGEs)

Over the period, *V. cholera* has evolved as one of the most successful pathogen in the history of mankind. To attain the fitness for survival, the pathogen has acquired a number of MGEs belongs to different classes such as prophages (CTXΦ, VGJΦ, RS1, TLCΦ), pathogenicity islands (VPI-1, VPI-2, VSP-1 & VSP-2) and integrative conjugative elements (ICEs) (Table 1). The key virulence factor of cholera, cholera toxin (CT) is encoded by the *ctxA* and *ctxB* genes that induces the secretion of fluid and electrolytes from the intestinal epithelial cells and causes the diarrhea. CT is acquired through irreversible integration of a single stranded DNA (+ssDNA) phage CTXΦ into the *dif* sites of either or both the chromosome of *V. cholerae* (Val et al., 2005). The second most crucial virulence factor of cholera pathogen, toxin-coregulated pilus (TCP), encoded by the genes present in the TCP locus of VPI-1, helps the pathogen in colonization in the gastrointestinal tract of the host and also act as a cell surface receptor for CTXΦ (Manning, 1997). This altogether suggests that the acquisition of the MGEs is the key for the fitness and evolution of the cholera pathogen for different pandemics. Therefore, understanding the role of MGEs acquired by pathogen over time is critical to develop strategies for managing the patient and the disease.

**Table 1:** Significance of integrative mobile genetic elements (IMGEs) in the *V. cholerae* pathogenesis and fitness.

**S. no**.	**IMGEs**	**Size (kb)**	**Cholera pathogenesis**				**Brief description and function**
			**Required for conversion of environmental non-pathogenic strains to pathogenic clones**	**Colonization**	**Role in toxin production**	**Overall enhanced pathogenicity**	
1.	CTXΦ	6.7	+	–	+	+	Integrates in the chromosome of *V. cholerae*, form stable lysogens and encodes cholera toxin Kimsey and Waldor, 1998
2.	RS1Φ	3	–	–	–	+	Carries gene for an anti-repressor RstC that effect CTXΦ replication Faruque et al., 2003a
3.	VGJΦ	7.5	–	–	–	+	Integrates into same *dif* site as CTXΦ Das et al., 2010
4.	TLCΦ	5.3	–	–	–	+	Generates a functional *dif* site in *dif* defective strains and facilitates stable integration of CTXΦ Hassan et al., 2010
5.	VPI-I	41.3	+	+	+	+	Encodes receptor for CTXΦ that also helps acquisition of the CTXΦ into the *V. cholerae*, bacterial colonization in the human-gut, regulates toxin production and helps the bacteria to achieve fitness in harsh environmental conditions Boyd et al., 2000
6.	VPI-II	57.3	–	–	–	+	Encodes neuraminidase which converts higher order sialogangliosides to GM-1 gangliosides, receptor for cholera toxin Jermyn and Boyd, 2002
7.	VSP-I	16	–	–	–	+	Encodes a putative XerCD like integrase Faruque and Mekalanos, 2003
8.	VSP-II	27	–	–	–	+	Encodes RNase H1 protein, a type IV pilus O'Shea et al., 2004

## Cholera and its Indispensible Association With VPI-1

**Figure 1:**
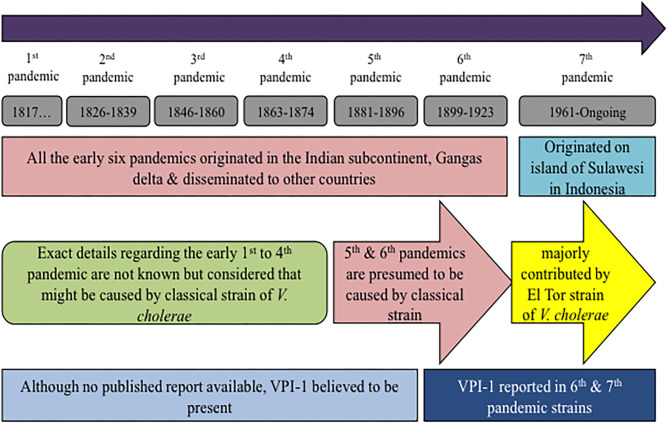
Chronological history of cholera pandemics and its association with VPI-1.

The first cholera pandemic was recorded in 1817 and seven pandemics have been recorded till to date (Figure 1) (Hu et al., 2016). The ongoing 7th pandemic was first evolved on the island of Sulawesi in Indonesia in 1961 (Karaolis et al., 1995). All of the seven cholera pandemics are caused by the O1 serotype of *V. cholerae*, except the spatial emergences of O139 Bengal in eastern part of India and Bangladesh in 1992 (Johnson et al., 1994). Interestingly, all of these pathogenic strains harbored VPI-1 as well as CTXΦ in their chromosomes (Li et al., 2003). Since VPI-1 encoded TcpA acts as a receptor for CTXΦ, sequential acquisition of VPI-1 and CTXΦ probably convert the environmental *V. cholerae* strains into toxigenic strains. Although no such experimental evidences or validation or natural phenomenon has been reported yet (Singh et al., 2001). But this is most accepted hypothesis across the scientific community that environmental *V. cholerae* strain may become pathogenic if it acquires VPI-1 and CTXΦ. Besides, loss of VPI-1 could revert a pathogenic strain into a non-pathogenic strain. Other than VPI-1 and CTXΦ, *V. cholerae* O1 El Tor biotype strains have acquired RS1 element (Choi et al., 2010) and two other pathogenicity-associated islands (VSP-1 & VSP-2). However, it is believed that RS1, VSP-1 & VSP-2 are not the pre-requisite for *V. cholerae* pathogenesis but involved in the fitness and robustness of 7th pandemic *V. cholerae* strains over the classical strain of O1 serotype.

## Vibrio Pathogenicity Island-1 (VPI-1)

The Vibrio pathogenicity island-1 (VPI-1) is a ~41.3 kb long DNA fragment present in all the epidemic strains including sixth-pandemic classical biotype and seventh-pandemic El Tor biotype strains. Previously, VPI-1 was experimentally demonstrated to be a filamentous bacteriophage (Karaolis et al., 1999). The authors reported that the cell-free preparations can transmit VPI-1 between *V. cholerae* strains by transduction. However, the protection of VPI-1 genomic DNA in a phage preparation from DNase and RNase treatment insisted the authors to conclude that the VPI-1 genes in the phage preparations are possibly wrapped with the protein coat TcpA. The authors further reported that replicative dsDNA genome of VPI-1 were detectable in toxigenic *V. cholerae* by DNA hybridization. However, no other laboratories were able to reproduce these findings (Faruque and Mekalanos, 2003). Nevertheless, the same group later on reported that VPI-1 is a pathogenicity island (Rajanna et al., 2003).

**Figure 2:**
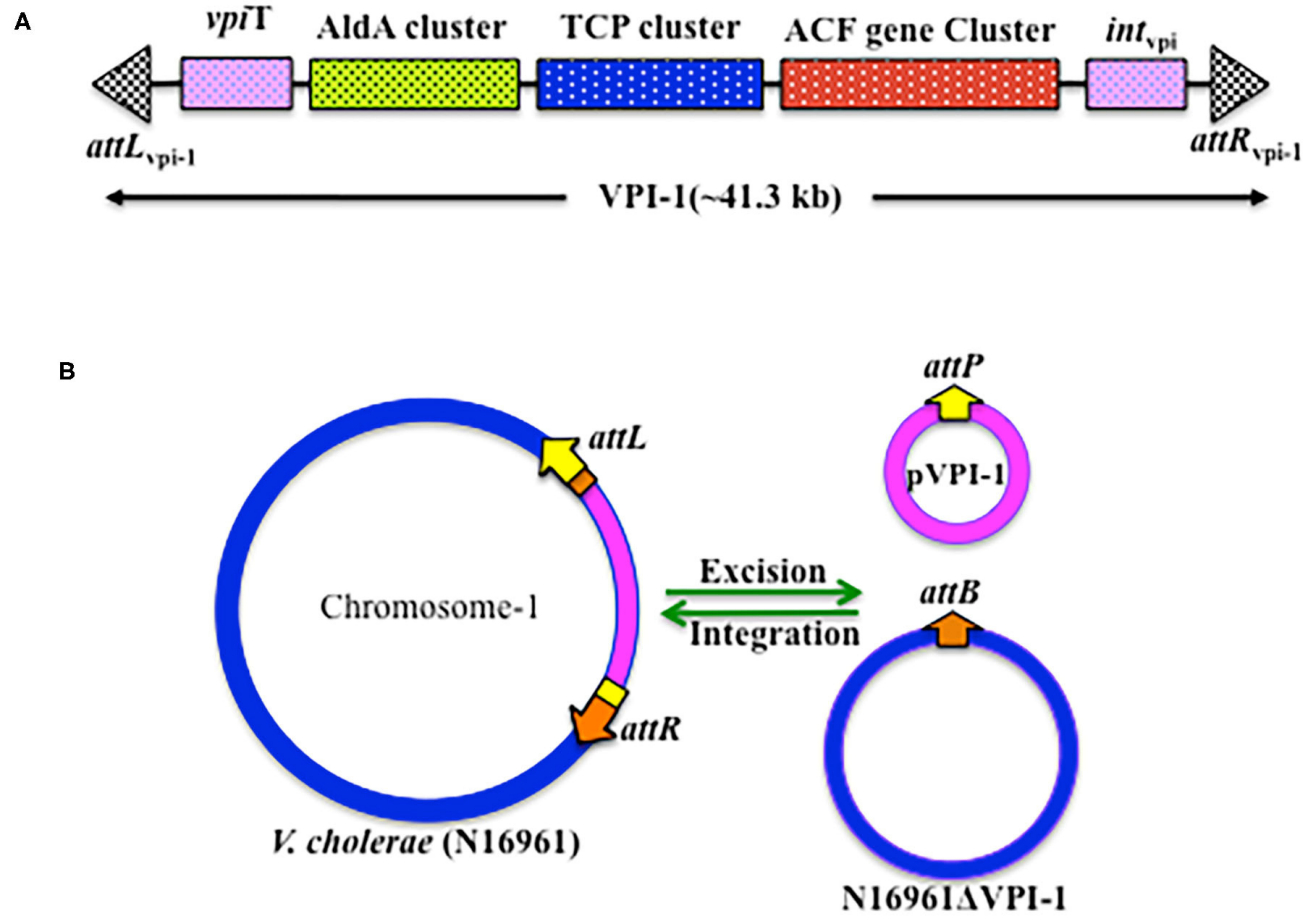
Genetic organization of VPI-1. **(A)** Schematic representation of the VPI-1 genetic element, **(B)** Schematic representation of the integration and excision mechanisms of VPI-1 genetic element.

In the whole genome sequenced reference *V. cholerae* strain N16961, VPI-1 harbors 31 genes (*VC0817* to *VC0847*) with known and unknown functions (Rajanna et al., 2003) (Table 2, Figure 2A). Like other genomic islands, VPI-1 has following typical characteristics:

(i) Sporadic distribution among environmental isolates
(ii) Distinct GC content (35% of the total in VPI-1 alone)
(iii) Flanked by direct repeat sequences (30–31 bp); one at left (*attL*) and another at right (*attR*) borders
(iv) Located downstream of a tmRNA locus
(v) Encodes two DNA mobility enzymes; one transposase [VpiT encoded by *vpiT* (Gene Map ID: VC0817)] and one integrase [Int_vpi_ encoded by *intV* (Gene Map ID: VC0847)]
(vi) Harbors a number of virulence associated and accessory colonization factors.

**Table 2:** Properties and function of VPI-1 encoded genes.

**Gene map ID**	**Gene symbol**	**Size**^**#**^ **(bp)**	**Mw** **(kDa)**	**Functions(s)**
VC0817	*vpiT*	984	38.2	Transposase, mediates integration, and excision Faruque et al., 2003b
VC0818	N/A	680	N/A	Pseudo gene, function N/A
VC0819	*aldA*	1,440	52.7	Expressed under the control of ToxR and may be associated with virulence Mishra et al., 2003
VC0820	*tagA*	3,042	115.9	A mucinase, involved in modification of intestinal cells surface during *V. cholerae* infection Hammer and Bassler, 2003
VC0821	N/A	4,501	N/A	Pseudo gene, function N/A
VC0822	N/A	3,331	N/A	Hypothetical protein, function N/A
VC0823	N/A	939	N/A	Hypothetical protein, function N/A
VC0824	*tpx*	495	17.9	A thiol-specific peroxidase that protect *V. cholerae* cells against oxidative stress Cha et al., 2004
VC0825	*tcpI*	1,863	69.0	Negatively regulates the *tcpA* expression in non-permissive conditions and promotes colonization in response to environmental single and also maximize *tcpA* expression in permissive growth conditions Harkey et al., 1994
VC0826	*tcpP*	666	25.7	Transcriptional activator of *toxT* Hase and Mekalanos, 1998
VC0827	*tcpH*	411	15.2	Required for stability of TcpP Carroll et al., 1997
VC0828	*tcpA*	675	23.2	Receptor for CTXΦ, helps in forming micro-colonies and play role in intestinal colonization of *V. cholerae* Rhine and Taylor, 1994
VC0829	*tcpB*	1,293	47.1	Mediates uptake of CTXΦ in to *V. cholerae* cells and also initiate the assembly of TCP Gao et al., 2016
VC0830	*tcpQ*	453	17.2	Required for the stability of TcpC and also help in outer membrane localization Bose and Taylor, 2005
VC0831	*tcpC*	1,470	53.8	Encode outer membrane lipoprotein required for pilus biogenesis and provide resistance from host complement system.
VC0832	*tcpR*	456	17.7	Helps in high osmolality tolerance in intestinal lumen and promotes colonization Tripathi and Taylor, 2007
VC0833	*tcpD*	837	31.7	TCP pilus biogenesis Parsot et al., 1991
VC0834	*tcpS*	459	17.3	Essential for colonization Davies et al., 2012
VC0835	*tcpT*	1,512	57.2	A cognate putative ATPase located on inner membrane, required for TCP biogenesis and also for all other TCP-mediated functions Chang et al., 2017
VC0836	*tcpE*	1,023	38.0	Probably involved in cholera toxin receptor (GM1) interaction Kolappan and Craig, 2013
VC0837	*tcpF*	1,017	38.1	A soluble protein, role in colonization Megli et al., 2011
VC0838	*toxT*	831	32.0	Master regulator of virulence associated genes, directly activates expression of cholera toxin and TCP, and also auto-regulates its own expression Schuhmacher and Klose, 1999
VC0839	*tcpJ*	762	29.3	Encode type IV prepilin peptidase, required for the processing of TcpA Kaufman et al., 1991
VC0840	*acfB*	1,880	69.1	Required for intestinal colonization, disruption of any of the four genes exhibits 10-fold decreases in colonization Hughes et al., 1995; Klose, 2000
VC0841	*acfC*	771	28.5	
VC0844	*acfA*	648	24.6	
VC0845	*acfD*	4,562	167.8	
VC0843	*tagE*	909	34.4	Probably encodes an endo-peptidase Almagro-Moreno et al., 2010
VC0846	N/A	1,701	N/A	Pseudo gene, function N/A
VC0847	*intV*	1,269	48.3	Integrase, mediates integration and excision Kumar et al., 2018

# Size and molecular weight are obtained from NCBI server and ExPASy portal of Swiss institute of Bioinformatics.

*N/A = Not available*.

The role of VPI-1 in the pathogenicity of cholera was identified while investigating the differences between pathogenic and non-pathogenic *V. cholerae* strains (Karaolis et al., 1998). Several proteins (ToxT, TcpA, TcpP, TcpH, ACFs gene cluster) encoded by the VPI-1 are crucial for *V. cholerae* pathogenesis and justified the name of the genomic island as Vibrio pathogenicity islands.

## Distribution of VPI-1 in *V. cholerae* Isolates

Genomic analyses of thousands of clinical *V. cholerae* isolates revealed that VPI-1 is widely conserved in the genome of all the epidemic and pandemic cholera pathogen (Mutreja et al., 2011; Domman et al., 2017; Weill et al., 2019). However, VPI-1 distribution is sporadic among nonO1-nonO139 environmental *V. cholerae* strain (Chun et al., 2009). Whole genome sequence analysis of environmental strains revealed that VPI-1 is absent in several isolates belonging to serogroup O37 (MZO-3), O39 (AM-19226), O12 (1587), O14 (MZO-2), O141 (V51), and O135 (RC 385). Interestingly, VPI-1 was detected in the nonO1-nonO139 environmental *V. cholerae* strains belonging to serogroup O141 (V51). The same group has also reported absence of VPI-1 in the genome of environmental O1 El Tor strains 12129 and TM11079-80 (Chun et al., 2009). As expected, the genome of all the VPI-1 negative *V. cholerae* strains are also negative for CTX-prophage. This finding indicates that VPI-1 is absolutely important for the conversion of a non-toxigenic strain to a toxigenic variant. In addition, VPI-1 encoded functions are also essential for colonization of cholera pathogen in the host intestine. Since, colonization and toxin are the sole components for development of cholera, VPI-1 is an indispensible component for the emergence and evolution of epidemic and pandemic *V. cholerae*.

## Mobility of VPI-1: Acquisition and Dissemination

VPI-1 has been reported to precisely excise from the *V. cholerae* chromosome and form an extra-chromosomal circular product (Rajanna et al., 2003) (Figure 2B). However, this study could not provide information about the loss frequency of VPI-1 from the genome of *V. cholerae*. To address this issue, Kumar et al. (2018) have engineered the genome of *V. cholerae* and developed a reporter strain to monitor the loss frequency of VPI-1 and isolated VPI-1 devoid clone from a mixed population of *V. cholerae*. Authors used an antibiotic resistance gene (*cat*) as selectable marker and a sucrose sensitive confirming counter selectable gene (*sacB*) to tagged the VPI-1 element. The engineered strain was used to measure loss frequency of VPI-1 in *in vitro* (test tube) and *in vivo* (rabbit ileal loop model) growth conditions. *V. cholerae* cell containing functional *sacB* in the genome is unable to grown in room temperature (~24°C) in the presence of excess sucrose (10–15%) sucrose in growth medium. Using the said vector, excision frequency of VPI-1 was investigated in the *in vitro*. VPI-1 was found highly stable in the animal model (excision frequency, ~10^−9^) compared to the *in vitro* laboratory growth condition (excision frequency, ~10^−4^) (Kumar et al., 2018).

The VPI-1 doesn't encode any known conjugative function. The pathogenicity island also has no *oriT* sequence. Thus, horizontal transmission of VPI-1 between different *V. cholerae* strains couldn't be through conjugation. Since *V. cholerae* is naturally competent (Meibom et al., 2005), the bacterium could uptake naked genomic DNA including VPI-1 through transformation. We believe that different *V. cholerae* serogroups living in the biofilms during aquatic and inflectional phases of its life cycle acquired MGEs through natural transformation. However, currently substantial experimental evidences are lacking to support this hypothesis. In another study, VPI-1 has also been shown to get transferred from *V. cholerae* O1 strain C6709 to VPI-negative *V. cholerae* isolates (468-83, GP6, V69, and 1528-79) by a generalized transducing phage CP-T1.This study proposed an alternative mode of dissemination of VPI-1 between *V. cholerae* strains (O'Shea and Boyd, 2002). Once inside the cell, both the tyrosine recombinases encoded by the VPI-1 helps the GI to integrate at the *prfC* locus by site-specific recombination. The reaction is reversible; hence the *attL* and *attR* sites generated due to integration of VPI-1 come together, possibly with the help of recombination directionality factors (RDFs), and excised from the chromosome.

Although VPI-1 has already been shown indispensable for *V. cholerae* pathogenicity, the molecular mechanisms and factors that modulate integration and excision of VPI-1 into chromosomes are still largely unknown.

## VPI-1 and Cholera Pathogenesis

**Figure 3:**
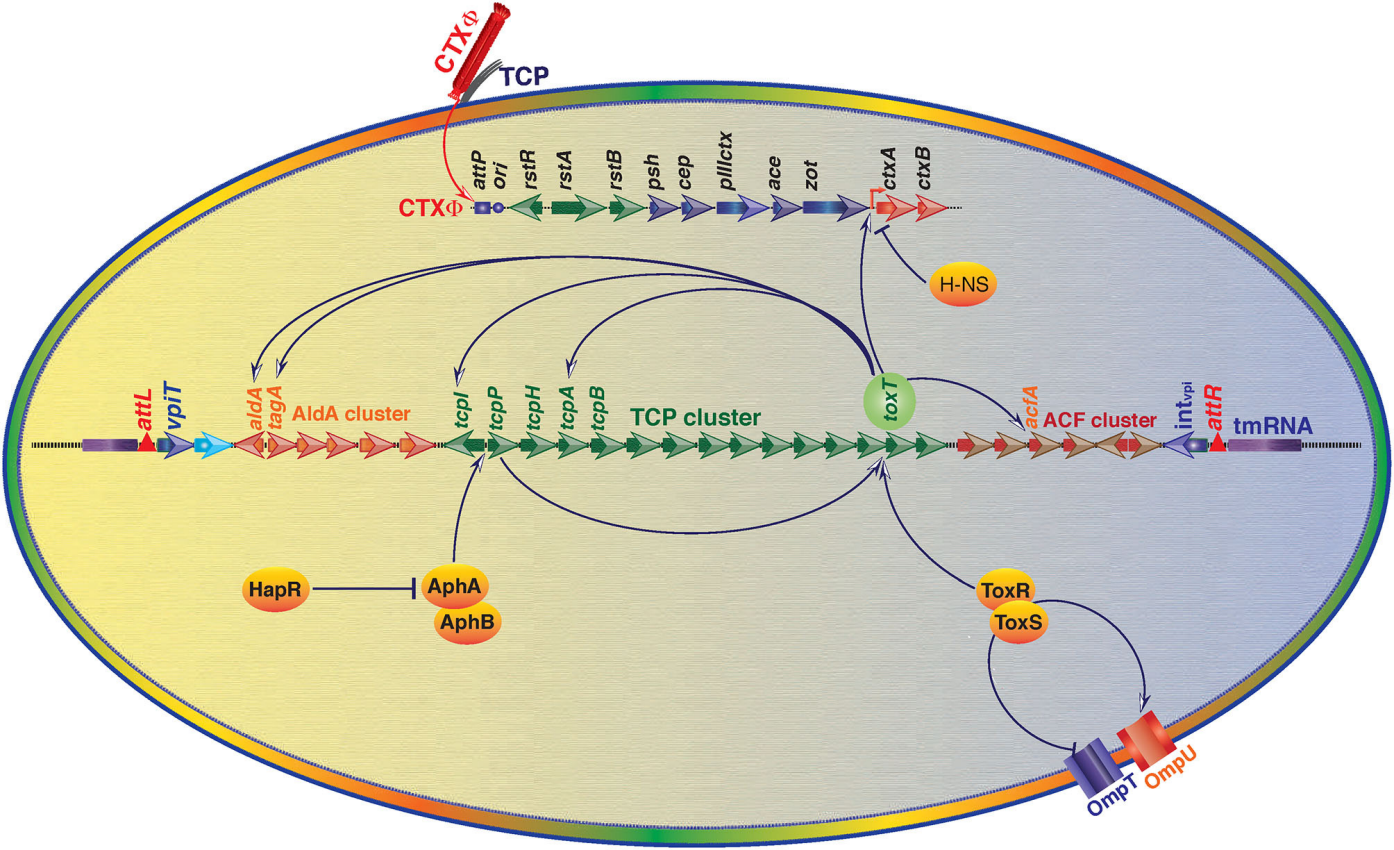
An overview of regulation of *V. cholerae* virulence gene expression through Vibrio Pathogenicity Island 1 (VPI-1) and core genome encoded functions. Transcriptional factor ToxT is the key positive regulator of cholera toxin (CT) encoding genes *ctxA* and *ctxB*. ToxT also positively modulates expression of toxin co-regulated pilus (TCP) genes and several other genes located in the VPI-1. ToxR-ToxS and AphA-AphB proteins positively regulate expression of *toxT*. ToxR-ToxS proteins also modulate expression of outer membrane proteins OmpU and OmpT. Histone-like nucleoid-structuring (H-NS) protein negatively regulates expression of CT. Transcriptional regulator HapR negatively regulates expression of AphA and AphB encoding gene expression. Positive regulation is depicted by arrowhead and T head depicts negative regulation.

Although CT is the crucial factor for developing cholera, but it is not the only virulence factor for developing the disease. There are several other virulence-associated factors (ToxT, TCP, AphAB, ToxRS) that also contribute substantially in cholera pathogenesis. Production of CT, and intestinal colonization of *V. cholerae* in the host intestine are pivotal for disease development (Krukonis et al., 2000). VPI-1 plays critical role in both the processes by encoding CT expression transcriptional factor ToxT and intestinal colonization factor TCP. Nevertheless, VPI-1 also plays crucial role in the acquisition of CT encoding gene through horizontal gene transfer. The detailed role of VPI-1 in cholera pathogenesis is as given below and depicted in Figure 3.

### Acquisition of CTXΦ in *V. cholerae*

Acquisition of CTXΦ is the first and critical step for the conversion of a non-toxigenic *V. cholerae* strain into toxigenic clone. The VPI-1 encoded TCP is a homopolymer of multiple subunits of major pillin protein, TcpA. After its production, TcpA trans-located to the cell surface and serve as the receptor for the CTXΦ (Lim et al., 2010). Once CTXΦ recognizes TcpA pili on the surface, it injects it's (+)ssDNA genome in to the host cytoplasm of the host cell(s) (Karaolis et al., 1999). The (+)ssDNA genome of CTXΦ either converted into replicative double stranded pCTXΦ or exploits the two tyrosine recombinases, XerC and XerD, for irreversible integration into chromosomal DNA (Boyd, 2010). XerC and XerD recombinases are conserved among the bacteria chromosomes and their native function is to resolve the chromosome dimers. XerC and XerD recognize 28 bp *dif* sequences as a substrate to resolve the chromosomal dimers before cell division (Das et al., 2013). The CTXΦ has two *dif* like attachment sequences, *attP1* and *attP2*, on its pCTXΦ genome. In (+) ssDNA genome of CTXΦ both the attachment sites are separated by 90 bp DNA sequences. The single stranded genome of CTXΦ forms intra-strand base pairing and developed a double-forked hairpin like structure. The stem of the hairpin harbors a functional attachment site (+)*attP* resembles with the *dif1* and *dif2* site of *V. cholerae* chromosomes (McLeod and Waldor, 2004). Due to similar DNA sequences between *dif* and (+)*attP*, the XerC and XerD recognizes the (+)*attP* as their binding substrate and mediate CTXΦ integration into the *dif* site by site specific recombination. Once the CTXΦ gets integrated into the chromosome, the host DNA replication machinery converts (+) ssDNA CTXΦ genome into double stranded. Once integrated, the dsDNA of prophage genome is unable to excise due to lack of two functional dif like sequences. This irreversible integration of CTXΦ into the chromosomal DNA of *V. cholerae* is one of the important events in the *V. cholerae* evolution as a pathogen. Tandemly integrated CTX-prophage can initiate rolling replication for production of new virion and disseminate to other *V. cholerae* cells. In addition to TcpA, several other VPI-1 functions including TcpB and TcpE helps acquisition and dissemination of CTXΦ (Gutierrez-Rodarte et al., 2019).

### Intestinal Colonization of *V. cholerae*

*V. cholerae* is the natural inhabitant of estuaries where it can survive as free-living cells or as biofilms. The stomach is not a suitable environment for *V. cholerae* to survive and multiply since, the bacterium is highly sensitive to low pH (Almagro-Moreno et al., 2015). However, on ingestion with contaminated food/water, *V. cholerae* strains have the ability to pass through such adverse environments and colonized in the small intestine of human gut and produce sufficient amount of cholera toxin to develop the clinical symptoms.

Historically chemotactic movement was proposed to be responsible for colonization of *V. cholerae* (Castro-Rosas and Escartin, 2002), however various reports later suggested that intestinal colonization of *V. cholerae* is the complex outcome of interplay of VPI-1, chromosomally encoded proteins and host factors that are involved in motility, chemo-taxsis, and penetration. During the initial stages of colonization that is at the proximal ends of small intestine, bacterium flagellum, and a few general (neuraminidase etc.) and *V. cholerae*-specific proteolytic enzymes (haemagglutinin/protease, Hap) helps in the movement and penetration of thick mucosal layer (Zhu and Mekalanos, 2003). TagA, a VPI-1 encoded metalloprotease, is known to modify the mucin glycoproteins that are attached to the host cell surface (Szabady et al., 2011). TagA is specifically expressed and secreted by the pathogen under virulence-inducing conditions. TagA has been reported to be positively coregulated along with TCP and other virulence genes and hence may potentially play critical role in colonization of the pathogen by facilitating the movement through the intestinal mucosa (Szabady et al., 2011). Along with a few pathogen-derived adhesin molecules (i.e., flagellin, Mam7, GbpA, OmpU, and FrhA etc.), TCP is also reported to help the pathogen to adhere to the epithelium and facilitate bacteria-bacteria interaction to form micro-colonies (Tam et al., 2007). The aberrant production or function of the TCP has been shown to result in reduced colonization in both humans and mice. In addition, other four VPI-1 encoded proteins called as accessory colonization factors encoded by *acfA, acfB, acfC, acfD* also play critical role in colonization (Everiss et al., 1994; Withey and DiRita, 2005; Valiente et al., 2018). Disruption of any of the four genes has been reported to cause ~10-fold decreases in colonization as compared to wild type.

### Clinical Appearance of the Disease

Once the *V. cholerae* colonized inside the gut it starts producing CT and other virulence factors. CT is A_1_B_5_ multimeric protein complex, where B-subunit binds to GM1 ganglioside receptor. The receptor-toxin complex is endocytosed to endoplasmic reticulum and A1-subunit of toxin activates adenylate cyclase that ultimately opens chloride ions channels (CFTR Cystic fibrosis trans-membrane conductance regulator) and causes the secretion of fluids and ions into the lumen of gut (Vanden Broeck et al., 2007). Clinical onset of cholera may be sudden or delayed, depending upon the inoculation size. But in general after the incubation period in range of 18 h to 5 days, clinical symptoms of diseases appeared that included secretion of voluminous stools, resembling like rice-water (Due to presence of mucus in the stool), abdominal discomfort, anorexia with or without vomiting (Sack et al., 2004). Although ~2–11% of the infected person with the El Tor and Classical strain of *V. cholerae*, respectively showed the severe clinical manifestation (Kaper et al., 1995). In the case of severe cholera, the rate of diarrhea reaches up to 500–1,000 ml/h, which leads to decrease in the turner pressure, low blood pressure, sunken eyes and wrinkled hand, and feet skin. Although cholera patients can be easily treated with simple electrolytes replacement therapy but severe patients of cholera may die within few hours if left untreated (Bhattacharya, 2003).

VPI-1 encoded transcriptional activator ToxT activates the transcription of CT; ToxT is a 32-kDa AraC family transcriptional activator of *ctxA*/*ctxB* as well as *tcpA, acfs, aldA*. It has two helix-turn-helix-motifs, an N-terminal dimerization and environmental-sensing domain, along with C-terminal DNA binding domain (Lowden et al., 2010). Once induced, the ToxT directly bind via direct repeats of the sequence TTTTGAT called as Tox boxes to the promoter of *ctxA, ctxB, tcpA, acfs, aldA*. However, configuration of tox boxes differs at different promoters. For example, binding to the promoter of *ctxA/B*, ToxT required minimum three direct repeats of Tox boxes (Prouty et al., 2005). For t*cpA* promoter, ToxT binds to two tox boxes arranged as a direct repeat between position −44 and −67. Whereas, for *acfA* and *acfD* promoters, ToxT binds to two tox boxes organized as an inverted repeat (Krukonis et al., 2000). In contrast to *tcpA, acfA*, and *acfD*, ToxT binds to a single tox box for *aldA* promoter. ToxT is located downstream of *tcpF* and auto-regulates its own transcription. A histone-like nucleoid structural protein (H-NS) that binds to DNA in sequence-independent manner at AT-rich sequences competes with ToxT binding promoters and causes the repression of virulence protein (Ayala et al., 2017). Probably this mechanism might help in the shutdown of virulence protein expression under non-permissive conditions. Same finding was supported with mutational studies; *h-ns* mutants are reported to have de-repressed expression of *toxT, ctxA*/*B*, and *tcpA* (Nye and Taylor, 2003). As compared to *tcpA* promoter, H-NS strongly binds to *ctxAB* promoter and subsequently strongly represses the transcription of *ctxAB* (Nye et al., 2000).

### Interaction Between VPI-1 and Chromosomally Encoded Function and Its Influence on *V. cholerae* Pathogenesis

The functioning of *V. cholerae* as a pathogen is strongly regulated by the function encoded by its core genome, genomic islands and intestinal environmental conditions in the host associated phage. In this review, we also included role of human intestinal environment in the *V. cholerae* pathogenesis. The human gut environment consists of a number of poorly characterized chemical components harboring thousands of host derived signaling molecules. In addition, microbiota residing in the intestine contribute in further complexity by producing and secreting several small molecules in the milieu. Auto-inducers that triggers the expression of number of virulence factors in *V. cholerae* are one of the important bacterial component that modulate disease severity and dissemination of bacteria from host to the environment (Higgins et al., 2007). A number of host and microbial origin molecule have been discovered to-date and majority of which have integrated molecular circuit with VPI-1 encoded transcriptional regulators that play role at various stages of cholera pathogenesis.

#### Core Genome Encoded HapR Repressed VPI-1 Encoded ToxT

The *aphA*/*hapR* mediated quorum sensing is the central mechanism that regulates the virulence-associated cascade in toxigenic *V. cholerae*. The low cell density of the pathogen in the gut is typically sensed by a membrane bound sensor kinase (the CqsS), based on the concentration of a cholera auto-inducer 1 (CAI-1). CAI-1 triggers the expression of *aphA*, which induces the expression of *tcpP* and *tcpH* (Haycocks et al., 2019; Herzog et al., 2019). This quorum sensing of *V. cholerae* modulates the virulence gene expression cascades for development of the disease and its severity. Besides at low cell density, CqsS activates LuxO (a sigma-54 dependent protein) that represses the expression of *hapR* which favors the bacterial growth and pathogenesis (Zhu et al., 2002). Whereas, at high cell density, LuxO is unable to activate the repressor protein of HapR, and hence it remains available to *aphA* promoter between −85 and −58 and repress its expression, which leads to deficiency of TcpP and TcpH. Ultimately because of this, ToxR/ToxS regulon becomes unable to activate the *toxT*; the master regulator of VPI-1 encoded factors and cholera toxin production (Kovacikova and Skorupski, 2002). Interestingly in *V. cholerae* strain N16961, *hapR* is not active due to a frame-shift mutation (Joelsson et al., 2006). Since, N16961 is a clinical isolate and it can develop typical disease phenotype in animal model indicating HapR is dispensable for disease development. For a clear understanding of the HapR mediated regulation, the interactions of HapR with the transcriptional factors AphAB has been depicted in the Figure 3. Some additional modulators like ToxT, H-NS, ToxR/S involved in the regulation of toxin production, colonization and disease development can compensate the absence of HapR in N16961.

#### ToxR and TcpP Mediated Activation of ToxT

ToxR is a 32.5-kDa inner trans-membrane transcriptional activator consisting of 294 amino acids. It has three functional domains: a 180 amino acids long cytoplasmic domain, 16 amino acids long trans-membrane domain, and 100 amino acids long peri-plasmic domain (Miller et al., 1987). ToxR helps *V. cholerae* in sensing external intestinal environmental conditions (like pH, bile, temperature) and facilitates bacterial adaptation (Childers and Klose, 2007). ToxR modulates the expression of CT, OmpU, OmpT, and ToxT by binding to their promoters and modulating their transcription (Provenzano and Klose, 2000). However, experimental data indicates that ToxR alone is unable to activate the ToxT expression and need another membrane bound protein TcpP. Possibly, ToxR act as an enhancer for TcpP at the *toxT* promoter. ToxR also interact with another co-transcribed protein, ToxS (Bina et al., 2003), which is required for the maximal transcriptional activation of ToxT regulated genes. Like ToxR, TcpP interacts with another protein, TcpH that is required for TcpP stability. Experimental data showed that when *V. cholerae* cells shifted from permissive to non-permissive growth conditions, TcpP gets degraded even in presence of TcpH suggesting the role of TcpP at the initial stage of virulence cascade as a switch of ToxT dependent virulence associated genes in permissive and non-permissive growth conditions (Raskin et al., 2020). Once the bacteria sense the gut environment through its quorum sensing, ToxR and TcpP binds to *toxT* promoter at −100 to −69 and at −51 to −32 region with respect to transcription start site, respectively and subsequently activates the expression *toxT*, the master regulator of *V. cholerae* pathogenesis and hence the disease.

#### VarS/VarA Mediated Activation of Virulence-Associated Genes

It is reported that VarS/VarA, two-component system acting downstream of ToxR/S signaling cascade, also independently controls the expression of ToxT and other virulence proteins in response to environmental signals. *V. cholerae* O395 *varA* mutant are reported to have reduced expression of *tcpA* and *ctxA* and *ctxB* (Tsou et al., 2011). VarS mutant of classical O395 and El Tor C6706 strains exhibit the decreased production of TcpA (134-fold), CT (2.5-fold), *ctxA* (1.75-fold) and *ctxB* (1.72-fold) as compared to wild types (Jang et al., 2011). Recently, downstream target of VarS/VarA system, PckA which is a key player in central carbon metabolism and affects the levels of many metabolic intermediates, has been shown to modulate HapR activity (Jang et al., 2010). Although more evidences are required, this supported to hypothesize that VarS/VarA system to be a way for *V. cholerae* to combine information about surrounding cell density and nutrient availability to fine-tune its gene expression profile precisely to the surrounding microenvironmental conditions.

## Genetic Variation in VPI-1, Among Various Clinical Isolates of *V. cholerae*

Classical strains of *V. cholerae* represent the remnant of the sixth-pandemics toxigenic clones, while El Tor and O139 Bengal represent the current seventh-pandemic toxigenic clones (Karaolis et al., 1995). Comparative studies of VPI-1 of sixth and seventh pandemics strains found that the GI integrate in the same locus of chromosome 1 of both the biotypes of O1 isolates. VPI-1 is very similar in both the biotypes and has similar numbers of ORFs. However, ~483 polymorphic nucleotides were reported between both the biotypes. The central region of VPI-1 that harbors gene for TcpI, TcpP, TcpH, and TcpA was found to have interestingly highest level of polymorphic nucleotides (Karaolis et al., 2001). Highest variation was observed for *tcpA*, gene encoding the type IV pilus that works as a receptor for CTXΦ, around 22.5% variation at nucleotide level and 16.9% at the protein level. The accessory colonization factor, AcfD that help in the intestinal colonization contained longer open reading frame in El Tor strain (Table 3). Despite the huge importance of VPI-1 encoded genes in the pathogenesis of *V. cholerae*, yet a detail comparative study of their contents including the characterization of genetic, proteomic, and phenotypic variations among the various pathogenic clones of *V. cholerae* is not available. Therefore, in order to understand the reason behind the disappearance of O1 classical strains and emergences of O1 El Tor strain and the temporal emergences of O139 Bengal in 1992, further investigations are of much demand.

**Table 3:** Genetic variation among the important genes of classical and El Tor strains.

**Gene**	**Percentage variation**
*tagD*	1.21%
*tcpP*	1.50%
*tcpH*	2.67%
*tcpB*	1.46%
*tcpQ*	1.98%
*tcpC*	2.17%
*tcpA*	22.5%

## Conclusion

Cholera, an acute gastrointestinal diarrheal disease caused by the *V. cholerae*, is still a major public health concern to many developing countries including India. Improved understanding of the cholera pathogenesis at molecular level is a pre-requisite for the development of appropriate strategies for disease management.

Acquisition of MGEs through HGT is among the most common approaches of the pathogens to achieve fitness and survival traits in hostile and/or changing environments. Two important virulence factors for cholera pathogenesis, CT and TCP, are part of two MGEs, CTXΦ and VPI-1, respectively. This indicates that understanding the MGEs biology, their integration mechanisms, stable inheritance and dissemination between bacterial species, may help in reducing the disease burden and development of novel therapeutics for treating the disease. To date, *V. cholerae* have acquired a number of MGEs (CTXΦ, VPI-1, VPI-2, RS1, VSP-1, & VSP-2) that help the pathogen survival under the changing environmental conditions and contribute in causing the pathogenesis. The VPI-1 is unique, as it has been involved in regulation of almost all the stages of cholera pathogenesis. First, the VPI-1 encoded TCP helps the CTXΦ to recognize the host bacterium and introduce their (+) ssDNA inside the host cell. The *V. cholerae* strains that lack CTXΦ are typically non-pathogenic. Then, VPI-1 encoded factors, including TCP, TagA, AcfA, AcfB, AcfC, and AcfD, facilitate the pathogenic bacterium to colonize in the human gut. The VPI-1 encoded ToxT induces the expression of CT, the most critical virulence factor of cholera pathogenesis and helps the pathogen to cause the disease. TCP, Tpx, and ToxT are also integral part of quorum sensing mechanisms that protects the pathogen from harsh micro-environmental conditions in the gut. Deregulation of VPI-1 or its encoded factor(s) have already been reported to negatively impact the ability of the pathogen to cause the disease. This altogether supports the role of VPI-1 as the master regulator of cholera pathogenesis and hence suggesting it as potential therapeutic target for disease control and/or management.

## Author Contributions

AK provided the general concept. AK and NK drafted the initial concept of manuscript. AK, NK, and BD wrote the manuscript. All the authors have seen and approved the final manuscript.

## Conflict of Interest

The authors declare that the research was conducted in the absence of any commercial or financial relationships that could be construed as a potential conflict of interest.
